# Anti-CD19 Monoclonal Antibodies: a New Approach to Lymphoma Therapy

**Published:** 2015

**Authors:** Fatemeh Naddafi, Fatemeh Davami

**Affiliations:** 1 *Pharmaceutical Sciences Research Center, Shahid Beheshti University of Medical Sciences, Tehran, Iran. *; 2 *Biotechnology Research Center, Pasteur Institute of Iran, Tehran, Iran. *

**Keywords:** Anti-CD19, monoclonal antibodies, B-cell lymphoma, anti-CD19 sFv, blinatumumab

## Abstract

CD19 is expressed on B- lineage cells and follicular dendritic cells and plays a key role in B cell malignancies and autoimmune diseases. Thus, it has been considered as potential target for several monoclonal antibodies (mAbs). For decades, chemotherapy has been known as one of the major antitumor therapies eradicating high proliferative tumor cells. But, anti- CD19 mAbs developed for treating CD19- positive lymphomas and autoimmune diseases would rank among the most novel area of research and development in the pharmaceutical industry. Moreover, several anti- CD19 mAbs are currently being tested in various clinical trials and this review provides an overview of the research accomplished so far.

## Introduction

**Fig. 1:**
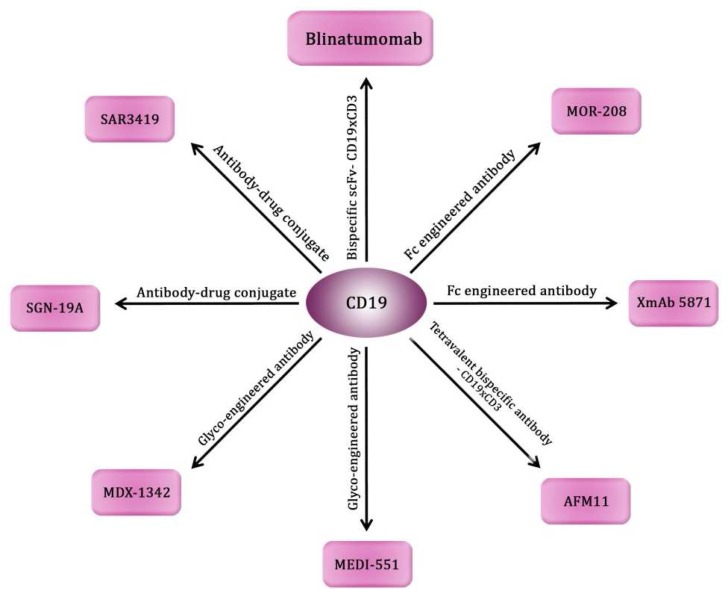
Anti CD19 mAbs types

**Fig. 2:**
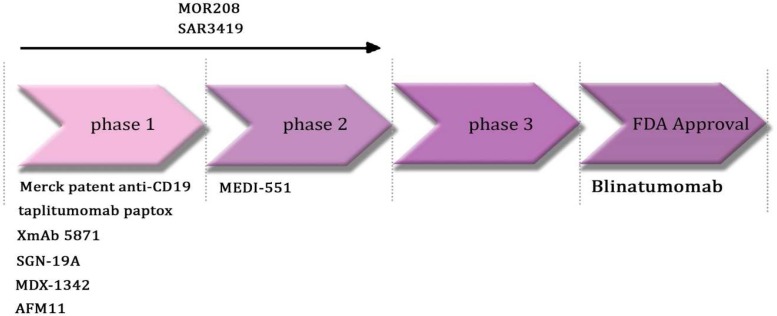
Anti CD19 mAbs in clinical trials

Acute leukemia is considered as the most common form of childhood malignancies (1), accounting for 20–30% of all pediatric malignan-cies depending on age (2). In the United States, about 2500 new patients are diagnosed annually; and 80% of these patients are B-lineage acute lymphoblastic leukemia (B-ALL) (1). In western countries, high incidence rates of non–Hodgkin's lymphoma (NHL) have been reported, and most of NHL subtypes are more prevalent among men than women (3). The fifth most common cancer in the United States is NHL and >66,000 patients have been diagnosed with NHL in 2008 (4). NHL is accounting for about 4% of all cancer diagnoses in incidence and deaths annually (5). CD19 is a 95- kDa transmembrane glycoprotein which belongs to the immunoglobulin (Ig) superfamily and can act as a central positive response regulator in B cells (6, 7). The CD19 has been expressed on the surface of leukemia cells in > 90% of cases with acute lymphoblastic leukemia (ALL). Moreover, it can be expressed on tumor cells of cases with both B-cell NHL and chronic lymphocytic leukemia (CLL). Thus, CD19 antigen is an ideal target for immunotherapy of B-cell malignancies (8, 9). CD19 can act as a B cell co-receptor in conjunction with both CD21 and CD81 (10). Genetic studies show that CD19 has a role in keeping a balance between immunity and autoimmunity (10). Although both CD19 and CD22 have their own regulatory network, they do not modulate B cell receptor (BCR) signals independently. CD19 modulates CD22 phoshoryla-tion by increasing Lyn kinase activity, while CD22 suppresses CD19 phosphorylation through SH-protein tyrosine phosphatase-1 (SHP-1). This ‘‘CD19/CD22 loop’’ could be directly related to an autoimmune phenotype in mice, so that it could be an attractive therapeutic target for regulating B cell signaling in autoimmune diseases (7). Although various antibody-based therapies targeting CD20 including rituxan® (rituximab) are on the market (11), anti CD19 monoclonal antibodies (mAbs) have lately been advanced into clinical trials. Many anti-CD19 IgG1 antibodies with cytotoxicity-improved Fcγ part and three drug conjugated antibodies including coltuximabravta-nsine (SAR3419), denintuzumab-mafodotin and taplitu-momabpaptox are now in phase 1/2 trials (5, 12) and the most advanced is the anti-CD19/CD3 BiTE antibody blinatumumab, which is in phase 1 trial in patients with NHL (13, 14) and was approved for Precursor B-cell acute lymphoblastic leukemia (B-cell ALL) on December 3, 2014 (12). Antibody drug conjugates (ADCs) comprise an antibody which is joined to a cytotoxic drug by a linker. ADCs could join the specificity of a monoclonal antibody (mAb) to the cytotoxicity of a small molecule drug .Thus, they are promising new therapeutic anticancer drugs. Currently, there are over 30 ADCs that are being investigated in early or late stage of clinical trials (15) (Table 1) (Fig. 1, 2). 

**Anti-CD19 monoclonal antibodies in clinical development**

**SAR3419**

SAR3419 (huB4-DM4), is a humanized IgG1 anti-CD19 antibody which is attached via a disulfide linker to a potent cell- killing maytansinoid derivative drug, DM4, which prevents both tubulin polymerization and microtubule assembly in the G2/M phase of the mitotic cycle and as a consequence, can cause apoptotic cell death (5). SAR3419 is in phase 1 trial for CD19+ NHL histological subtypes and it indicates encouraging activity in indolent and aggressive NHL with 33% error at the maximum-tolerated dose (MTD). Moreover, the lack of remarkable myelosuppression makes it an attractive mAb to be combined with chemotherapy drugs. Both pre and post treatment have revealed DM4 assembly in tumor and a decline in CD19 protein expression level. These data demonstrate promising clinical activity of SAR3419 in NHL (16). Thus, it could be a promising drug in patients who were refractory to rituximab (17). Furthermore, in preclinical studies, SAR3419 has been identified as a novel and well-tolerated therapeutic agent in B-cell NHL (4). It has been reported that SAR3419 delays the progression of four CD19+ B-cell precursor-ALL and three of three mixed lineage leukemia-ALL xenografts, causing objective responses in all but one xenograft. But it cannot be effective against T-lineage ALL xenografts. SAR3419 can exert remarkable efficacy against chemoresistant B-cell precursor-ALL xenografts over a high dose range (10-fold) and delayed vincristine/dexamethasone/L-asparaginase (VXL)-induced leukemia progression in two chemore-sistant xenografts by up to eighty two days (18). In prolonged treatment which is following remission induction with VXL, SAR3419 may inhibit disease recurrence into hematolymphoid without central nervous system involvement. These data confirm that the involvement of SAR3419 into remission induction protocols improve the outcome for both pediatric and adult CD19+ ALL (18).

**MOR-208**

MOR-208 (XmAb5574) is in phase 2 and was developed by Xencor and MorphoSys AG as a therapeutic drug for NHL and ALL (12). XmAb-5574 is a humanized mouse antibody with an engineered Fc domain with two amino acid substitutions S239D and I332E that increases its cytotoxic potency by binding to Fcγ receptors IIIa on immune cells and reduces binding to FcγRIIb. Both *in vitro* and *in vivo* studies have shown that XmAb5574 increases antibody dependent cell-mediated cytotoxicity (ADCC) 100-fold to 1,000-fold which confirms its anticancer effects against a wide range of B-lymphoma (19). In another study, the administration of XmAb5574 to cynomolgus monkeys demonstrated a decline of B cells in the lymphoid tissues. Moreover, a decrease in peri-pheral NK-cell levels and loss of the maximal rate of B-cell have been found. It has also been munifested that XmAb5574 couples to monkey CD19 and demonstrates increased binding to FcγRs (20). The XmAb5574-dependent ADCC can be modulated by natural killer (NK) cells via a granzyme B–dependent mechanism (19). XmAb5574 can functionally activate both NK-cell lysosomal associated membrane protein-1 (CD107a) production and IFN-γ secretion. Erk1/2 belongs to the mitogen-activated protein kinase family that is serine-threonine kinases and its phosphorylation is essential to FcγRIIIa-induced granule exocytosis in NK cells. NK cells stimula-tion in the presence of XmAb5574 leads to up- regulation of Erk1/2 phosphorylation (19).

**Table 1:** A brief overview of anti – CD 19 mAbs

Antibody	Internal name	Antigen	Company	Phase	Condition	Antibody type
Blinatumomab	AMG103	CD19,CD3	Amgen	Approved 1	ALL NHL	×
Coltuximabravtansine	SAR3419, huB4-DM4	CD19	ImmunoGen Inc. Sanofi-aventis	2 1	ALL, NHL	Humanized mouse antibody drug conjugate/IgG1
MOR208	XmAb 5574	CD19	Morphosys AG, Xencor Inc.	2 1	ALL, NHL CLL	Humanized mouse antibody
MEDI-551	MEDI-551	CD19	Medimmune	2	B-cell malignancies, CL L, Multiple Myeloma, Scleroderma	Humanized mouse antibody
Denintuzumabmafodotin	SGN-19A, SGN-CD19A, BU12	CD19	Seattle Genetics	1	NHL	Humanized undefined source antibody drug conjugate/IgG1
Merck patent anti-CD19	B4, DI-B4	CD19	Merck Serono	1	B-cell malignancies	Humanized mouse antibody
Taplitumomabpaptox	Taplitumomabpaptox	CD19	National Cancer Institute	1	B-cell malignancies	Mouse antibody drug conjugate/IgG1
XmAb 5871	XmAb 5871	CD19	Amgen, Xencor Inc.	1	Autoimmune Diseases	Humanized undefined source antibody
MDX-1342	MDX-1342	CD19	Medarex	1	CLL,Rheumatoid Arthritis	Human-from transgenic mouse antibody
AFM11	AFM11	CD19, CD3	Affimed	1	NHL	Human bispecific antibody

CD: cluster of differentiation; ALL: acute lymphoblastic leukemia; BiTE: bispecifc T cell engager; CLL: chronic lymphocytic leukemia; NHL: non-Hodgkin lymphoma.

**MEDI-551**

MEDI-551 can target CD19 on B cells and entered phase 2 trial in 2011. MEDI-551 was developed by Medimmune (12). A novel afucosylated anti-human (hu) CD19 mAb, MEDI-551 has been produced with high affinity to human FcγRIIIA and mouse FcγRIV and increased ADCC. MEDI-551 was highlighted to be effective at much lower mAb concentrations than the fucosylated parental mAb anti-CD19-2 in *in vitro* ADCC assays with B-cell lines. Moreover, it has been proven that the afucosylated CD19 mAb MEDI-551 reduced B cells from normal donor peripheral blood mononu-clear cell samples in an autologous ADCC assay (21). Both blood and tissue B cells in human CD19/CD20 double transgenic (Tg) mice have been in lower concentrations than that of the positive control mAb rituximab. Furthermore, macro-phage-mediated phagocytosis and complement-dependent cytotoxicity might have a role in depletion with rituximab in huCD19/CD20 Tg mice (21). B-cell-depleting activity of MEDI- 551 has been demonstrated in both *in vitro* and *in vivo* studies and could be a promising novel drug for the treatment of both B-cell malignancies and auto-immune diseases (21). MEDI-551 is effective in tumor growth inhibition in multiple preclinical mouse xenograft models and has profound activity in combination with the CD20 mAb rituximab (22). It has been found that MEDI -551 treatment of severe combined immunodeficiency (SCID) mice engrafted with human pre-B cells caused long term animal survival and as a consequence decreased disease burden in blood, liver and bone marrow. These studies indicate that anti-CD19 antibodies strongly recruit immune cells to precursor-B ALL cells and can enter to early phase trials in pre-B acute lymphoblastic leukemia (23). Single-agent activity with a manageable toxicity profile has been found in CLL cases treated in phase 1/2 trial of MEDI-551. A phase 2 study of MEDI-551 combined with bendamustine in relapsed CLL cases (NCT01466153) is investigating clinical response to both MEDI-551 and chemotherapy (24).

**SGN-CD19A**

Since CD19 has been expressed in most B-cell NHL patients, denintuzumabmafodotin (SGN-CD19A) can be a novel antibody-drug conjugate consisting of a humanized anti-CD19 mAb attached to the microtubule-disrupting agent monomethyla-uristatin F (MMAF) through a maleimidocaproyl linker. SGN-CD19A has indicated signs of clinical activity with an objective response rate (ORR) of 40% (8 of 20 cases) and an observed complete response rate of 30% (6 of 20 cases). No dose-limiting toxicity was found in tested doses and further studies are needed to determine the optimal dose of SGN-CD19A (25).

**XmAb5871**

XmAb5871 is a humanized Fc engineered antibody attached to FcgRIIb with approximately 400-fold higher affinity compared with its native IgG1 Fc (26). XmAb5871 which was generated by both Amgen and Xencor Inc, entered phase 1 trial for autoimmune diseases in 2011 (12). The Fc receptor (FcγRIIb) could suppress B cell responses when coengaged with BCR. Thus, it is identified as a target for the treatment of new autoimmune diseases. It has been shown that the coengagement of BCR and FcγRIIb through the Fc-engineered anti-CD19 XmAb5871 inhibits humoral immune responses (26). XmAb5871 declines both ERK and AKT activation, cell proliferation, cytokine, and IgG synthesis that are induced by both BCR and TLR9 signals. Moreover, XmAb5871 suppresses differentiation of citrullinated peptide-specific plasma cells from rheumatoid arthritis cases. Thus, XmAb5871 can play potential role in the suppression of pathogenic B cells in autoimmune diseases (26-28). It has been proven that XmAb5871 causes phosphorylation of the ITIM of FcgRIIb and inhibits BCR-induced calcium mobilization, proliferation, and costimulatory expression of human B cells from both healthy donors and systemic lupus erythematosus (SLE) cases (29), and also B cell proliferation stimulated by lipopolysaccharides (LPS), interleukin 4 (IL-4), or B-cell activating factor (BAFF). Furthermore, XmAb5871 inhibits humoral immunity against tetanus toxoid and declines serum IgM, IgG, and IgE levels in SCID mice which are engrafted with either SLE or healthy human peripheral blood mononuclear cells (PBMCs) (29). Treatment with XmAb5871 led to an increase in the survival of mice engrafted with PBMCs from a unique SLE donor. It has been evidenced that coengagement of both FcgRIIb and BCR complex could not increase B cell depletion in human PBMCs cultures or in mice. Thus, stimulation of the FcgRIIb inhibitory pathway in activated B cells can be an attractive therapeutic approach for the treatment of systemic lupus erythematosus and other autoimmune diseases (29).

**MDX-1342**

MDX-1342 is a human anti- CD19 antibody which entered phase1 trial for chronic lymphocytic leukemia in 2008 and was supported by Medarex (Bristol-Myers Squibb). A phase 1 trial for rheuma-toid arthritis was completed in June 2013 (12). MDX-1342 is able to bind to human CD19-expressing cells, which is similar to its fucosylated parental antibody. MDX-1342 has an increased affinity for both FcγRIIIa-Phe158 and FcγRIIIa-Val158 receptors. Moreover, an increased effector cell function has been indicated by enhanced efficacy in ADCC assay. Furthermore, its low nanomolar binding to cynomolgus monkey CD19 and enhanced affinity for cynomolgus monkey FcγRIIIa is found. *In vivo* studies have indicated that MDX-1342 in cynomolgus monkeys leads to potent B-cell depletion. Thus, MDX-1342 can be considered as a B-lymphocyte depletive therapy for both malignancies and autoimmune diseases (30). MDX-1342 has comparable effects with rituximab for *in vivo* B-cell depletion. When these mAbs are administered as a single dose, both extent and duration of blood B-cell depletion is comparable, but the onset of depletion with rituximab is more rapid (21). 

**AFM11**

AFM11 is a human bispecific antibody which entered phase 1 trial in 2014 and was developed by Affimed Therapeutics AG as a therapeutic drug for NHL (12). AFM11 recruits T cells to kill CD19- positive cells and *in vitro* studies indicate a much higher potency and efficacy of tumor cell lysis by AFM11 compared with a bispecific tandem scFv. In addition, *in vivo* analysis of AFM11 shows a dose-dependent growth inhibition of Raji tumor cells and a single dose of AFM11 shows similar efficacy as 5 daily injections (31). It has been found that AFM11 can activate T cells only in the presence of CD19- positive cells. In peripheral PBMCs cultures, AFM11 has been seen to induce both CD69 and CD25 expression, T cell proliferation, and secretion of IFN-γ, TNF-α, IL-2, IL-6, and IL-10. Thus, AFM11 could be a promising drug for the treatment of CD19- positive tumors (31). 

**Blinatumomab**

Blinatumomab is a 55 kDa fusion protein which is composed of two single chain antibodies (scFvs) to both CD19 and CD3 that are connected via a flexible linker (32-34). Blinatumomab was developed by Micromet and entered phase 1 for NHL in 2004 (12). It was approved by FDA for precursor B-cell ALL therapy on December 3, 2014 This bispecific T-cell-engaging antibody, can couple polyclonal T cells to CD19-expressing B cells and bring both T cells and malignant B cells in close proximity. As a consequence, it induces killing of malignant B cells (34). It has been found that the cytotoxicity modulated by both blinatumomab and rituximab leads to a potent activation of pro-caspases 3 and 7 in target cells, which could result in the induction of granzyme-mediated apoptotic cell death (35). Although the comparative studies proved that the cytotoxic activity caused by blinatumomab with T cells is considerably more than ADCC by rituximab, the combination of both rituximab and blinatumomab increases the activity of rituximab, particularly at low effector-to-target cell ratios and at low antibody concentration (35). In an *in vitro* study, blinatumo-mab was cocultured with NALM-6, the CD19-expressing pre-B lymphoma cell line, and CD8-expressing T cells. Consequently, it increased cell contact with both T and NALM-6 cells and enhanced apoptosis and lysis of NALM-6 cells (33, 34). Cell lysis was not found in CD19- negative malignancies particularly, an erythroleukemia cell line 28 and a colon cancer cell line 29, indicating that CD3 binding by blinatumomab alone is not sufficient to cause T- cell activation. In the absence of major histocompatibility complex class I expression, T-cell-mediated cell lysis has been maintained (33, 34). Blinatumomab recruits all cytotoxic T cells for lysis of cancer cells. In NHL cases who received doses as low as 0.005 mg/m^2^/day, an elimination of target cells in blood was reported. Both partial and complete tumor regressions were first found at a dose level of 0.015 mg and a tumor regression was seen in all seven cases who received a dose level of 0.06 mg. Furthermore, blinatumomab could cause tumor cell clearance from bone marrow and liver (36). Blinatumomab-expanded T cells (BET) have been proven to have normal expression of the synapse inhibitors CD272 and CD279 in comparison with starting T cells and could be cytotoxic against CD19+ targets in the presence of blinatumomab *in vitro*. It has been found that a combination of BET and blinatumomab have remarkable therapeutic effect in a systemic human diffuse large B lymphoma model in NOD-SCID mice. Thus, BET can be identified as a therapeutic tool for immunoreconstitution of heavily immunosuppres-sed CLL cases and, in combination with blinatumo-mab, could be considered as anti-tumor immuno-therapy (37). After the start of infusion in patients treated with blinatumomab, a rapid drop in peripheral T cell counts has been reported and also an expansion above baseline counts was found during the treatment. In addition, binding of blinatumomab to both T and Raji lymphoma cells could lead to CD69 up-regulation on activated T cells surface (38). After a median of 398 days follow-up of posttransplant relapsed pediatric cases with B-precursor ALL, who were given blinatumomab as a four week continuous intra-venous infusion at a dosage of 5 or 15 μg/m^2^/day, the probability of hematologic event-free survival has been reported to be 30%. Moreover, major toxicities were grade three seizures in one case and grade three cytokine release syndrome in two cases. It has been found that blinatumomab can induce molecular remission in pediatric cases with posttransplant relapsed B-precursor ALL (39). It has been illustrated that blinatumomab can induce an 80% minimal residual disease (MRD) response rate in a phase 2 study of B-lineage ALL cases with persistent/ relapsed MRD (40). A recent study has indicated that after a median of 33 months follow-up, the hematologic relapse- free survival of the entire evaluable cohort of 20 cases was 61%. In addition, the hematologic relapse-free survival rate of 9 cases that were given allogeneic hematopoietic stem cell transplantation after blinatumomab treatment was 65%. In conclusion, blinatumomab could induce long-term complete remission in B-lineage ALL cases with either persistent or recurrent MRD. Furthermore, blinatumomab is known as an efficacious and well-tolerated therapy in MRD-positive B-lineage ALL cases after intensive chemotherapy. Coengagement of both T cells and blinatumomab could eradicate chemotherapy-resistant tumor cells that drive clinical relapse (40).

## Conclusion

The CD19 antigen can be expressed on the surface of leukemia cells in patients with ALL and NHL. Thus, it could be the main target for anti-CD19 mAbs. Therapy with mAbs has been developed in the last decade, due to their mechanisms of action and side-effects. Since bispecific antibodies including blinatumumab, which brings a cytotoxic T cell and the cancer target cell in close proximity and effectively eradicates tumor cells, has been investigated and indicated high response rates in cases with both NHL and ALL, it is considered as promising B-lineage anti-tumoral drug tested in various clinical trials for NHL and ALL and is the first anti-CD19 approved drug for precursor B-cell ALL in December 2014. Anti-CD19 monoclonal antibodies that are in phase 1/2 clinical trials are expected to be advanced into clinical development in the future.

## Conflict of Interests

The authors declared no conflict of interests.
